# The energy-level crossing behavior and quantum Fisher information in a quantum well with spin-orbit coupling

**DOI:** 10.1038/srep22347

**Published:** 2016-03-02

**Authors:** Z. H. Wang, Q. Zheng, Xiaoguang Wang, Yong Li

**Affiliations:** 1 Center for Quantum Sciences, Northeast Normal University, Changchun 130117, China; 2 Beijing Computational Science Research Center, Beijing 100094, China; 3 School of Mathematics and Computer Science, Guizhou Normal University, Guiyang 550001, China; 4 Zhejiang Institute of Modern Physics, Department of Physics, Zhejiang University, Hangzhou 310027, China; 5 Synergetic Innovation Center of Quantum Information and Quantum Physics, University of Science and Technology of China, Hefei 230026, China

## Abstract

We study the energy-level crossing behavior in a two-dimensional quantum well with the Rashba and Dresselhaus spin-orbit couplings (SOCs). By mapping the SOC Hamiltonian onto an anisotropic Rabi model, we obtain the approximate ground state and its quantum Fisher information (QFI) via performing a unitary transformation. We find that the energy-level crossing can occur in the quantum well system within the available parameters rather than in cavity and circuit quantum eletrodynamics systems. Furthermore, the influence of two kinds of SOCs on the QFI is investigated and an intuitive explanation from the viewpoint of the stationary perturbation theory is given.

In semiconductor physics, the spin-orbit coupling (SOC), which is available to generate the so-called spin-orbit qubit^1^, is widely studied in the field of both spintronics^2^ and quantum information^3^. In the low dimensional semiconductor, there exist two types of SOCs, that is the Rashba SOC which comes from the structure inversion^4^ and the Dresselhaus SOC which comes from the bulk-inversion asymmetry^5^. In general cases, the two types of SOCs coexist in a material^6^.

The spin properties of the electron(s) in a semiconductor material have been studied widely and it shows that some novel features emerge when the SOC is present. Among the various properties, the ones for the ground state play crucial roles. In this paper, we study the ground state of the electron in a semiconductor quantum well, which is subject to the Rashba and Dresselhaus SOCs as well as a perpendicular magnetic field. The Hamiltonian in this two-dimensional structure can be mapped onto a Hamiltonian describes a qubit interacting with a single bosonic mode, where the spin degree of freedom of the electron serves as the qubit and the orbit degree of freedom serves as the bosonic mode^7^,^8^. Furthermore, the Rashba SOC contributes to the rotating wave interaction and the Dresselhaus SOC contributes to the counter-rotating wave interaction. When the strengths and/or the phases of the two types of SOCs are not equal to each other (this is the usual case in realistic material), the mapped Hamiltonian is actually an anisotropic Rabi model^9^ in quantum optics.

With the available parameters in quantum well systems, it will undergo the energy-level crossing between the ground and first excited states as the increase of SOC strength. This kind of energy-level crossing will induce a large entanglement for the ground state, and have some potential applications in quantum information processing. Also, the steady state of the system when the dissipation is present is also affected greatly by the energy-level crossing. Although the same form of Hamiltonian (i.e., anisotropic Rabi Hamiltonian) can also be achieved in cavity and circuit quantum electrodynamics (QED) systems, such a crossing would not occur since the related coupling between the bosonic mode and the qubit is too weak. In this paper, we analytically give the crossing strength of Rashba SOC in which the energy-level crossing occurs when the Dresselhaus SOC is absent. Furthermore, we study the crossing phenomenon numerically when both of the two kinds of SOCs are present.

The energy-level crossing behavior in our system is similar to the superradiant quantum phase transition in the Dicke model^10^, where the quantum properties (e.g., the expectation of photon number in ground state) are subject to abrupt changes when the coupling strength between the atoms and field reaches its critical value^11^. Recently, it is found that the quantum Fisher information (QFI) is a sensitive probe to the quantum phase transition^12^,^13^. This inspires us to investigate the relation between the QFI and energy-level crossing in our system. The QFI, as a key quantity in quantum estimation theory, is introduced by extending the classical Fisher information to quantum regime, and can characterize the sensitivity of a state with respect to the change of a parameter. The QFI is also related to quantum clone^14^,^15^ and quantum Zeno dynamics^16^. In our system, we find that there exists an abrupt change in the QFI at the crossing point, so that the QFI can be regarded as a signature of the energy-level crossing behavior in quantum well system. Furthermore, the QFI increases with the increase of the Dresselhaus SOC. As the increase of Rashba SOC strength, the QFI nearly remains before the crossing but decreases monotonously after it. Actually, the QFI has a close connection with the entanglement^17^, and can be used to detect the entanglement^18^, so our results can be explained from the viewpoint of the entanglement and intuitively understood based on the stationary perturbation theory.

## Results

### System and Hamiltonian

We consider an electron with mass *m*_0_ and effective mass *m* moving in a two-dimensional *xy* plane, which is provided by a semiconductor quantum well. The electron is subject to the Rashba and Dresselhaus SOCs, and a static magnetic field in the positive *z* direction, i.e., 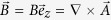. The Hamiltonian of the system is written as^7^


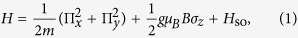


where 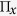 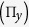 is the x- (y-) direction component of the canonical momentum 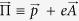 with 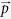 the mechanical momentum and 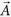 the vector potential. *g* is the Lande factor, and 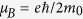 is the Bohr magneton, *σ*_*x*,*y*,*z*_ are the Pauli operators. Here, *ħ* is the Plank constant and 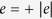 is the electronic charge.

The last term in Hamiltonian (1), representing the SOCs, can be divided into two terms *H*_so_ = *H*_*R*_ + *H*_*D*_, where


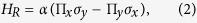



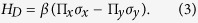


The Hamiltonian *H*_*R*_ and *H*_*D*_ represent the Rashba^4^ and Dresselhaus SOCs^5^ term, respectively. *α* and *β*, which are real and in units of velocity, describe the related strengths of the two types of SOCs and are determined by the geometry of the heterostructure and the external electric field across the field, respectively^19^.

Since we consider that 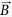 is along the positive direction of *z* axis, it is natural to choose the vector potential as 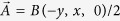. By defining the operator^7^


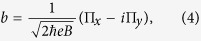


it is easy to verify that [*b*, *b*^†^] = 1, so *b* (*b*^†^) can be regarded as a bosonic annihilation (creation) operator.

In terms of *b* and *b*^†^, the Hamiltonian can be re-written as


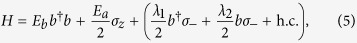


where *σ*_±_ = *σ*_*x*_ ± *iσ*_*y*_, and the parameters are calculated as


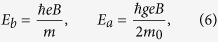



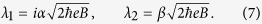


Thus, we have mapped the Hamiltonian in quantum well with SOCs onto a standard anisotropic Rabi model^9^,^20^,^21^,^22^,^23^ which describes the interaction between a qubit and a single bosonic mode. Here the spin and orbit degrees of freedom serve as the qubit and bosonic mode respectively. In the language of quantum optics, the first two terms in Eq. (5) are the free terms of the boson mode with eig-energy *E*_*b*_ and the qubit with the transition energy *E*_*a*_ respectively. The first term as well as its hermitian conjugate in the braket in Eq. (5) represents the rotating-wave coupling with strength 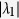 and the second term as well as its hermitian conjugate represents the counter-rotating coupling with strength 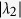, the relative phase between these two kinds of coupling is *π*/2 [see Eq. 7(b)]. Actually, such kind of mapping from spintronics to quantum optics can also be performed when an additional harmonic potential is added to confine the spatial movement of the electron^8^,^24^.

In our system, both of the bare energies of the qubit and bosonic mode as well as their coupling strength can be adjusted by changing the amplitude of the external magnetic field, so that the coupling strength can be either smaller or even (much) larger than the bare energies, this fact will lead to some intrinsic phenomena, such as the energy-level crossing^25^,^26^, which will be studied in what follows.

### The energy-level crossing

To investigate the energy-level crossing in our system, we begin with the case without the Dresselhaus SOC analytically, and the crossing behavior for the full Hamiltonian will be numerically discussed subsequently.

When the Dresselhaus SOC is absent (*β* = 0, then *λ*_2_ = 0), the mapped Hamiltonian reduces to the exact Jaynes-Cummings (JC) Hamiltonian where the excitation number is conserved. The eigen-state without excitation is 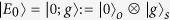, which represents that the orbit degree of freedom is in the bosonic vacuum state and the spin degree of freedom is in its ground state (actually, is the spin-down state because the magnetic field is in +*z* direction in our consideration). The corresponding eigen-energy is *E*_0_ = −*E*_*a*_/2. In the subspace with only one excitation, the pair of dressed states are


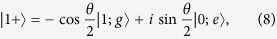



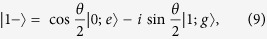


and the corresponding eigen-energies are


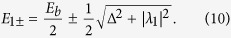


In the above equations, we have defined 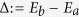, and


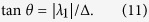


Using the above results, it is shown that the ground state of the system is either the separated state 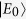 when *E*_1−_ > *E*_0_, or the entangled state 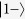 when *E*_1−_ < *E*_0_. A simple calculation gives the crossing Rashba SOC strength 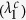 which distinguishes the entangled from separated ground state as


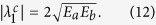


On the other hand, when the Dresselhaus SOC is present, the mapped Hamiltonian yields an anisotropic Rabi Hamiltonian, in which the rotating-wave term and the counter-rotating-wave term coexist. In this case, the conservation of the excitation is broken, that is, 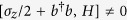. The analytical solution of quantum Rabi model (*λ*_1_ = *λ*_2_) was originally obtained by Braak^27^ and was developed to the case of anisotropic Rabi model (*λ*_1_ ≠ *λ*_2_)^9^. Their results however are based on a composite transcendental function defined by power series, and are difficult to extract the fundamental physics.

To deal with the counter-rotating-wave coupling term approximately, we now resort to a unitary transformation to the Hamiltonian *H*^20^,^28^,^29^,^30^,


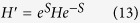


with


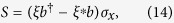


where the parameter *ξ* is to be determined. The detailed calculation is given in the method, and the final effective Hamiltonian is approximately obtained as *H*′ ≈ *H*_*a*_ + *H*_*b*_, where


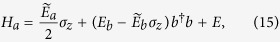



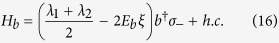


with 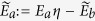, 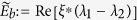, and 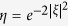.

It is obvious that the approximate Hamiltonian *H*′ has a similar form as the JC Hamiltonian, and the eigen-energies in the zero- and one-excitation subspace are obtained as


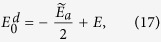



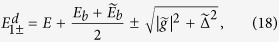


where


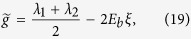



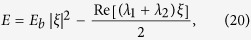



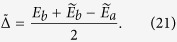


**Figure 1:**
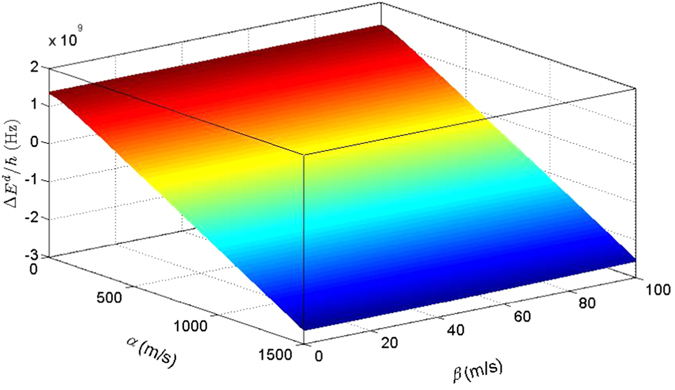
The energy gap as a function of *α* and *β*. The parameters are chosen as *g* = 1.52, *m*_0_ = 9 × 10^−31^ kg, *m* = 0.15*m*_0_, *B* = 0.01 T. Under these parameters, we will have 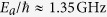, 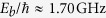, and 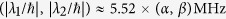.

The energy-level crossing then occurs when 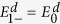. In Fig. 1, we plot the energy gap 
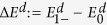
 as a function of *α* and *β*.

As shown in Fig. 1, for small *α*, 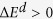, and the ground state is


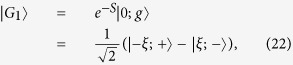


where 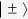 are the eigen-states of the Pauli operator *σ*_*x*_ satisfying 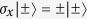, and 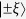 are the bosonic coherent states with amplitudes ±*ξ*.

As the increase of *α*, the energy-level crossing occurs, that is, Δ*E*^*d*^ becomes negative, and the ground state becomes


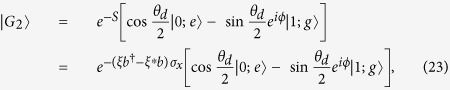


where


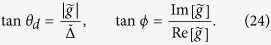


### Quantum Fisher information of the ground state

From now on, the QFI of the ground state is adopted to further characterize the energy-level crossing behavior and we will study its dependence on *α* and *β* in details. We will also give an intuitive explanation about the obtained results based on the stationary perturbation theory.

The so-called quantum Cramér-Rao (CR) inequality, obtained by extending the classical CR inequality to quantum probability and choosing the quantum measurement procedure for any given quantum state to maximize the classical CR inequality, gives a bound to the variance 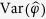 of any unbiased estimator 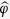
31,


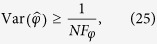


where *F*_*φ*_ is the QFI and *N* is the number of independent measurements. A larger QFI corresponds to a more accurate estimation to the parameter *φ*. Moreover, the QFI is connected to the Bures distance^31^ through


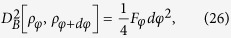


where the Bures distance is defined as 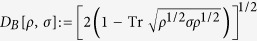.

For an arbitrary given quantum state, its QFI can be determined by the spectrum decomposition of the state. Fortunately, for a pure quantum state 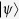, its QFI with respect to the parameter *φ* has a relatively simple form as^32^,^33^,^34^,^35^


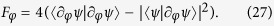


In what follows, we will consider the external magnetic field *B* as the parameter to be estimated, and study the corresponding QFI for the ground state of the system under consideration.

Before the energy-level crossing occurs (Δ*E*^*d*^ > 0), the ground state of the system is 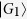 in Eq. (22). Its corresponding QFI with respect to the external magnetic field *B* is given as


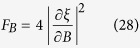


after some straightforward calculations.

After the energy-level crossing occurs (Δ*E*^*d*^ < 0), the ground state is 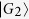 in Eq. (23). The expression of the related QFI of the ground state with respect to *B* is tedious and we will only give the numerical results here.

**Figure 2:**
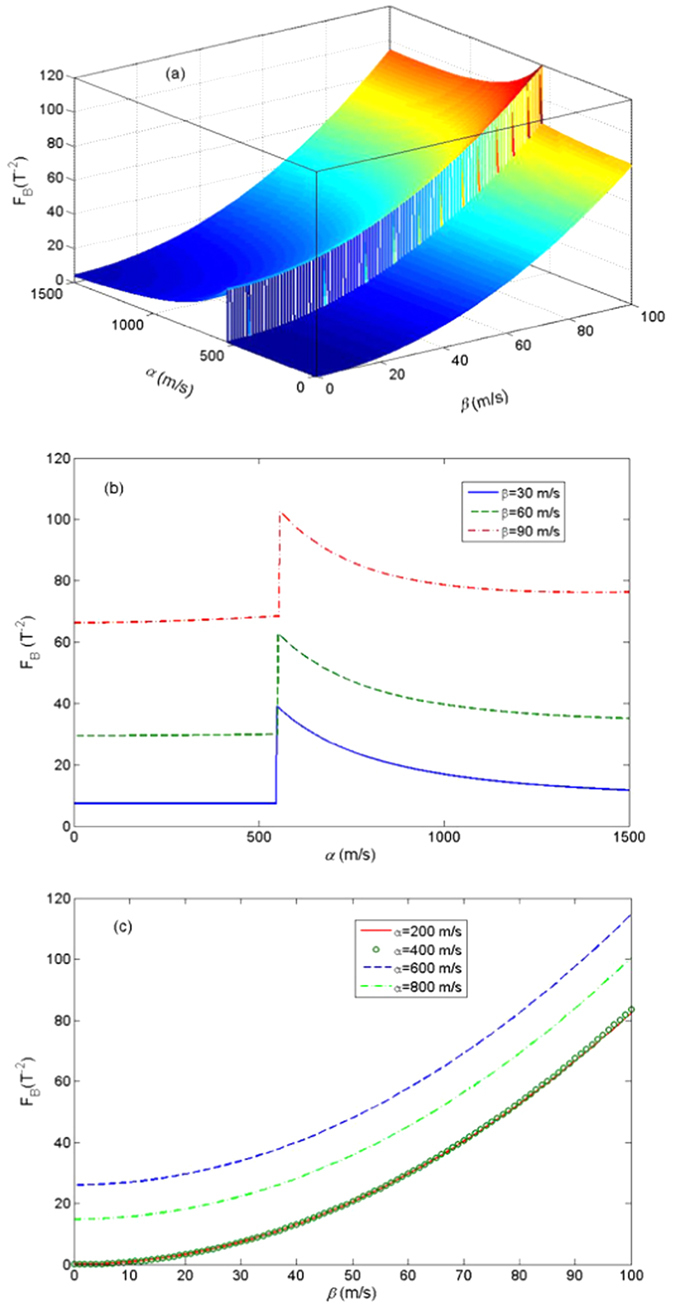
(**a**) The QFI *F*_*B*_ of the ground state with respect to the external magnetic field *B*, as a function of *α* and *β*. (**b**) *F*_*B*_ as a function of *α* for different *β*. (**c**) *F*_*B*_ as a function of *β* for different *α*. The other parameters are the same as those in Fig. 1.

In Fig. 2(a), we plot the QFI as a function of *α* and *β*. It obviously shows that the QFI undergoes a sudden change when the energy-level crossing occurs. Therefore, the QFI of the ground state can be regarded as a witness of the energy-level crossing behavior.

Furthermore, in Fig. 2(b), we plot the QFI as a function of *α* for different values of *β*. On one hand, the QFI nearly keeps constant when *α* approaches the crossing point from small values, and decreases monotonously when *α* surpasses the crossing value *α*^*c*^ (*α*^*c*^ ≈ 550 m/s within our chosen parameters and it corresponds to 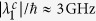). On the other hand, a larger *β* will lead to a larger QFI, which implies a more precise measurement about the magnetic field. This result is also demonstrated in Fig. 2(c), where the QFI is plotted as a function of *β* for different values of *α*. It shows that the curves for *α* = 200 m/s and *α* = 400 m/s, which are both below the crossing values, coincide with each other. As for the values above the crossing point, we observe a decreasing behavior of QFI as the increase of *α*, for example, the QFI for *α* = 600 m/s is larger than that for *α* = 800 m/s as shown in Fig. 2(c).

**Figure 3:**
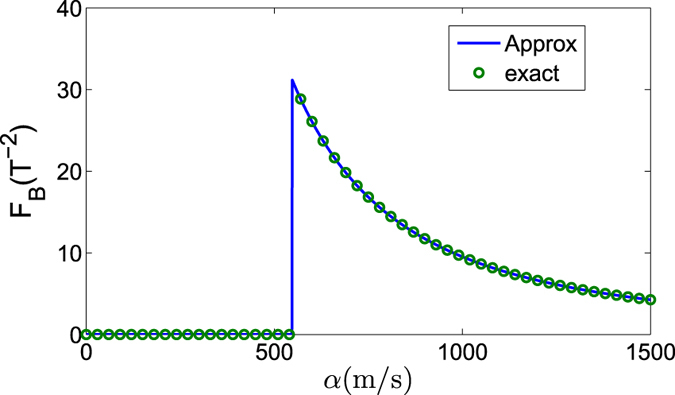
The QFI *F*_*B*_ of the ground state with respect to the external magnetic field *B*, as a function of *α* when *β* = 0. The other parameters are same as those in Fig. 1. Here, we use the solid line to represent the approximate result (which is obtained based on the approximate Hamiltonian *H*′ ≈ *H*_*a*_ + *H*_*b*_) and the empty circles to represent the exact result.

The above results can be naturally recovered to the case of *β* = 0 (i.e., only the Rashba SOC is present). In Fig. 3, we plot the QFI of the ground state for *β* = 0 based on the approximate Hamiltonian *H*′ ≈ *H*_*a*_ + *H*_*b*_ after the transformation (see the empty circles). Actually, in the case of *β* = 0, we can also obtain the QFI of the ground state exactly according to the Hamiltonian *H* without the transformation. As shown in the above discussions, in this case the ground state is the separated state 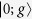 [the entangled one 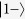 in Eq. (9)] before (after) the energy-level crossing occurs. The corresponding QFI with respect to *B* is *F*_*B*_(*β* = 0) = 0 for 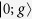 or 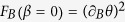 for 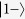 according to the general formula of QFI in Eq. (27). This exact result of the QFI *F*_*B*_(*β* = 0) is plotted in Fig. 3 (see the solid line). We find that these approximate and exact results agree well with each other and the QFI shows a sensitive dependence on *α* after the energy-level crossing occurs.

The dependence of QFI on the strengths of the Rashba and Dresselhaus SOCs can be explained from the viewpoint of the stationary perturbation theory qualitatively as what follows. In our consideration, the strength of Dresselhaus SOC is much weaker than that of the Rashba SOC and the bare energy of spin/orbit degree of the freedom, so it can be regarded as a perturbation. In this sense, the mapped Hamiltonian [Eq. (5)] can be divided into *H* = *H*_0_ + *H*_*I*_, where the un-perturbation part is


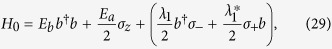


and the perturbation part is


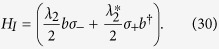


For small *α* or 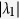, the ground state of *H*_0_ is 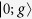 which is independent of the field *B* and yields a zero QFI. The perturbation part, which is contributed from the Dresselhaus SOC, mixes the state 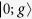 with 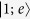, yields an entangled ground state and gives a non-zero *β* 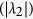 dependent QFI. It is obvious that the Dresselhaus SOC will enhance the entanglement, so that the QFI also increases as *β* becomes larger.

For large *α* or 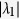, the energy-level crossing occurs, and the ground state of *H*_0_ becomes the wave function given in Eq. (9), which is an entangled state, yields a non-zero QFI. Furthermore, the entanglement decreases (increases) with the increase of *α* (*β*), and so the QFI behaves in a similar way.

## Method

By applying the unitary transformation defined in Eq. (13), the Hamiltonian *H*′ is obtained as *H*′ = *H*_*a*_ + *H*_*b*_ + *H*_*c*_ where


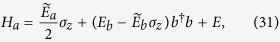



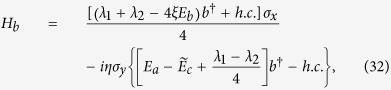



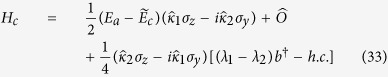


with 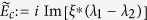. Here 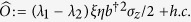 corresponds to the “two-photon” processes. We have also defined 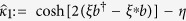 and 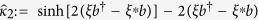, which are in the order of 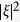 and 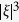, respectively.

When 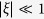 (which is valid as shown in what follows), we will neglect *H*_*c*_ and then *H*′ ≈ *H*_*a*_ + *H*_*b*_. For further simplicity, we can properly choose *ξ* to eliminate the counter-rotating-wave terms in *H*_*b*_, for which *ξ* satisfies


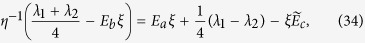


and then


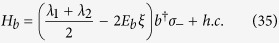


Thus, the approximate Hamiltonian *H*′ ≈ *H*_*a*_ + *H*_*b*_ can be solved exactly.

**Figure 4:**
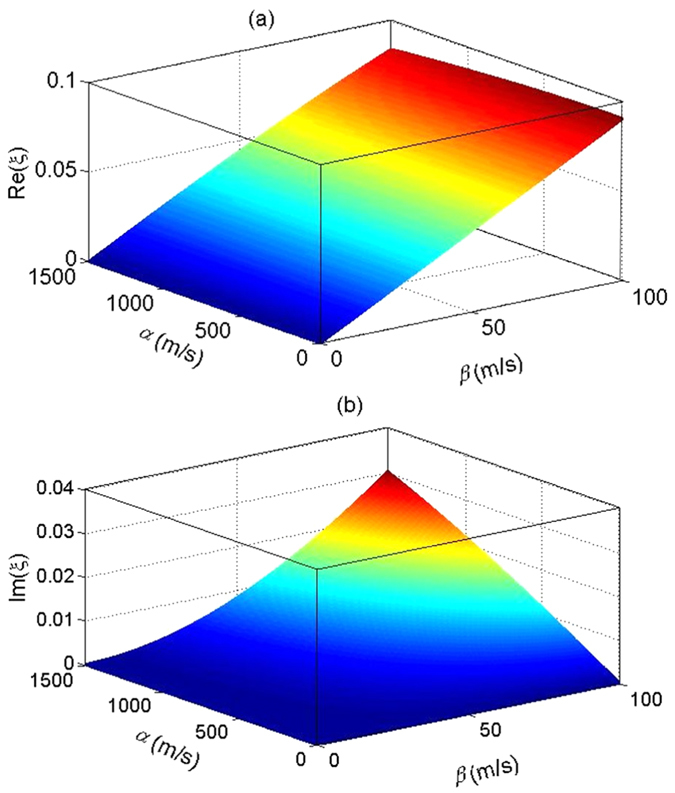
The real and imaginary parts of *ξ* as functions of *α* and *β*. The parameters are same as those in Fig. 1.

We note that, when both *λ*_1_ and *λ*_2_ are real numbers, *ξ* is also real, then our transformed Hamiltonian and the equation *ξ* satisfies coincide exactly with those in a recent paper^20^. However, as shown in Eq. (7), here *λ*_1_ is a pure imaginary number and *λ*_2_ is a real number, so that *ξ* is a complex number. We numerically solve Eq. (34), and plot the real and imaginary parts of *ξ* in Fig. 4 as functions of *α* and *β* for *B* = 0.01 T. It is obvious that 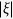 is indeed much smaller than 1, so that we can safely neglect the effect of *H*_*c*_ which is at least in the order of 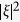.

## Discussion

We have shown that the Hamiltonian in the two-dimensional quantum well system can be mapped as an anisotropic Rabi model. Actually, the anisotropic Rabi model can also be realized in various systems, e.g., in the cavity or circuit QED systems. In a typical cavity QED system, in which the atom interacts with the optical cavity mode, the frequencies of the atomic transition and the cavity mode are of the order of 10^4^ − 10^5^ GHz, and the coupling strength reaches hundreds of MHz^36^. In a typical circuit QED system, where the artificial atom (superconducting qubit) couples to the transmission line resonator, the frequencies of the qubit and the resonator are about several GHz, and the coupling strength can be realized by hundreds of MHz^37^,^38^. In these two kinds of systems, which motivate many research interests during the past decades, the energy-level crossing can hardly occur since the coupling strength is not strong enough. However, the energy-level crossing can be available in the realistic quantum well material. Taking the AlAs material as an example, the Lande factor is *g* = 1.52, the mass of electron is *m*_0_ = 9 × 10^−31^ kg, and the effective mass is *m* = 0.15*m*_0_ 39. When the quantum well is subject to a magnetic field *B* = 0.01 T in +*z* direction, we will have 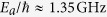, 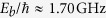, and 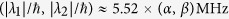. When choosing the parameters *α* in the order of hundreds of m/s and *β* in the order of tens of m/s, which can be achieved easily with the recent available experimental techniques^7^, the coupling strength could be in the same order or even larger than the energies *E*_*a*_ and *E*_*b*_. Therefore, the two-dimensional quantum well system provides a promising platform to simulate the energy-level crossing behavior and related phenomenon.

We would like to note that here we just focus on studying the QFI of the ground state (instead of excited states) to witness the energy-level crossing behavior. This is due to the fact that the property of the ground state is the simplest one to investigate but demonstrates clearly the energy-level crossing behavior in the quantum system under consideration, and the ground state is relative to the steady state when the dissipation is considered. This is similar to the case of the studies of quantum phase transition in the Dicke model, where only the QFI of the ground state is enough to probe the quantum phase transition^12^.

In summary, we investigate the energy-level crossing behavior and the QFI of the ground state with respect to the external magnetic field in a semiconductor quantum well. The Hamiltonian of the system with the Rashba and Dresselhaus SOCs simultaneously is mapped onto an anisotropic Rabi model in quantum optics. We find that although the mapped Hamiltonian is similar to that in cavity and circuit QED systems, the energy-level crossing behavior only occurs in our current system with the available parameters. As a probe of the energy-level crossing in our system, we discuss the QFI of the ground state and find that the QFI exhibits different dependencies on the strengths of the Rashba and Dresselhaus SOCs and has a sudden jump when the crossing happens. Based on the stationary perturbation theory, we give an intuitive explanation to the results.

## Additional Information

**How to cite this article**: Wang, Z. H. *et al.* The energy-level crossing behavior and quantum Fisher information in a quantum well with spin-orbit coupling. *Sci. Rep.* **6**, 22347; doi: 10.1038/srep22347 (2016).

## References

[b1] Nadj-Perge S., Frolov S. M., Bakkers E. P. A. M. & Kouwenhoven L. P. Spin-orbit qubit in a semiconductor nanowire, Nature (London) 468, 1084 (2010). 21179164 10.1038/nature09682

[b2] Zutic I., Fabian J. & Das Sarma S. Spintronics: Fundamentals and applications, Rev. Mod. Phys. 76, 323 (2004).

[b3] Awschalom D. D., Samarth N. & Loss D. Semiconductor Spintronics and Quantum Computation (Springer-Verlag, Berlin, 2002).

[b4] Bychkov Y. A. & Rashba E. I. Oscillatory effects and the magnetic susceptibility of carriers in inversion layers, J. Phys. C 17, 6039 (1984).

[b5] Dresselhaus G. Spin-Orbit coupling effects in Zinc blende structures, Phys. Rev. 100, 580 (1955).

[b6] Voskoboynikov O., Lee C. P. & Tretyak O. Spin-orbit splitting in semiconductor quantum dots with a parabolic confinement potential, Phys. Rev. B 63, 165306 (2001).

[b7] Schiemann J., Egues J. C. & Loss D. Variational study of the *v* = 1 quantum Hall ferromagnet in the presence of spin-orbit interaction, Phys. Rev. B 67, 085302 (2003).

[b8] Debald S. & Emary C. Spin-Orbit-driven coherent oscillations in a few-electron quantum dot, Phys. Rev. Lett. 94, 226803 (2005). 16090425 10.1103/PhysRevLett.94.226803

[b9] Xie Q.-T. *et al.* Anisotropic Rabi model, Phys. Rev. X 4, 021046 (2014).

[b10] Dicke R. H. Coherence in spontaneous radiation processes, Phys. Rev. 93, 99 (1954).

[b11] Nagy D., Konya G., Szirmai G. & Domokos P. Dicke-model phase transition in the quantum motion of a Bose-Einstein condensate in an optical cavity, Phys. Rev. Lett. 104, 130401 (2010). 20481867 10.1103/PhysRevLett.104.130401

[b12] Wang T.-L. *et al.* Quantum Fisher information as a signature of the superradiant quantum phase transition, New J. Phys. 16, 063039 (2014).

[b13] Salvatori G., Mandarino A. & Paris M. G. A. Quantum metrology in Lipkin-Meshkov-Glick critical systems, Phys. Rev. A 90, 022111 (2014).

[b14] Yao Y. *et al.* Multiple phase estimation in quantum cloning machines, Phys. Rev. A 90, 022327 (2014).

[b15] Song H., Luo S., Li N. & Chang L. Comparing quantum cloning: A Fisher-information perspective, Phys. Rev. A 88, 042121 (2013).

[b16] Smerzi A. Zeno Dynamics, Indistinguishability of state and entanglement, Phys. Rev. Lett. 109, 150410 (2012). 23102286 10.1103/PhysRevLett.109.150410

[b17] Pezze L. & Smerzi A. Entanglement, nonlinear dynamics and the Heisenberg limit, Phys. Rev. Lett. 102, 100401 (2009). 19392092 10.1103/PhysRevLett.102.100401

[b18] Li N. & Luo S. Entanglement detection via quantum Fisher information, Phys. Rev. A 88, 014301 (2013).

[b19] Rashba E. I. Electron spin operation by electric fields: spin dynamics and spin injection, Physica E 20, 189 (2004).

[b20] Shen L.-T. *et al.* Ground state of the asymmetric Rabi model in the ultrastrong coupling regime, Applied Physics B 117, 195 (2014).

[b21] Zhang G. & Zhu H. Analytical solution for the anisotropic Rabi model: effects of counter-rotating terms, Sci. Rep. 5, 8756 (2015). 25736827 10.1038/srep08756 PMC4348629

[b22] Wang Y. & Haw J. Y. Bridging the gap between the Jaynes-Cummings and Rabi models using an intermediate rotating wave approximation, Phys. Lett. A 379, 779 (2015).

[b23] Baksic A. & Cuiti C. Controlling discrete and continuous symmetries in “Superradiant” phase transitions with circuit QED systems, Phys. Rev. Lett. 112, 173601 (2014). 24836245 10.1103/PhysRevLett.112.173601

[b24] Zhao N., Zhong L., Zhu J.-L. & Sun C. P. Spin entanglement induced by spin-orbit interactions in coupled quantum dots, Phys. Rev. B 74, 075307 (2006).

[b25] Ashhab S. Superradiance transition in a system with a single qubit and a single oscillator, Phys. Rev. A 87, 013826 (2013).

[b26] Bulaev D. V. & Loss D. Spin relaxation and countercrossing in quantum dots: Rashba versus Dresselhaus spin-orbit coupling, Phys. Rev. B 71, 205324 (2005).

[b27] Braak D. Integrability of the Rabi model, Phys. Rev. Lett. 107, 100401 (2011). 21981483 10.1103/PhysRevLett.107.100401

[b28] Lü Z. G. & Zheng H. Quantum dynamics of the dissipative two-state system coupled with a sub-Ohmic bath, Phys. Rev. B 75, 054302 (2007).

[b29] Gan G. J. & Zheng H. Dynamics of a two-level system coupled to a quantum oscillator: transformed rotating-wave approximation, Eur. Phys. J. D 59, 473 (2010).

[b30] Ai Q., Li Y., Zheng H. & Sun C. P. Quantum anti-Zeno effect without rotating wave approximation, Phys. Rev. A 81, 042116 (2010).

[b31] Braunstein S. L. & Caves C. M. Statistical distance and the geometry of quantum states, Phys. Rev. Lett. 72, 3439 (1994). 10056200 10.1103/PhysRevLett.72.3439

[b32] Braunstein S. L., Caves C. M. & Milburn G. J. Generalized uncertainty relations: Theory, examples, and Lorentz invariance, Ann. Phys. (NY) 247, 135 (1996).

[b33] Zhong W. *et al.* Fisher information under decoherence in Bloch representation, Phys. Rev. A 87, 022337 (2013).

[b34] Zhang Y. M., Li X. W., Yang W. & Jin G. R. Quantum Fisher information of entangled coherent states in the presence of photon loss, Phys. Rev. A 88, 043832 (2013).

[b35] Zheng Q., Ge L., Yao Y. & Zhi Q.-J. Enhancing parameter precision of optimal quantum estimation by direct quantum feedback, Phys. Rev. A 91, 033805 (2015).

[b36] Grimsmo A. L. & Parkins S. Cavity-QED simulation of qubit-oscillator dynamics in the ultrastrong-coupling regime, Phys. Rev. A 87, 033814 (2013).

[b37] Chiorescu I. *et al.* Coherent dynamics of a flux qubit coupled to a harmonic oscillator, Nature (London) 431, 159 (2004). 15356624 10.1038/nature02831

[b38] Murali K. V. R. M. *et al.* Probing decoherence with electromagnetically induced transparency in superconductive quantum circuits, Phys. Rev. Lett. 93, 087003 (2004). 15447217 10.1103/PhysRevLett.93.087003

[b39] Rodriguez M. V. & Nazmitdinov R. G. Model for spin-orbit effects in two-dimensional semiconductors in magnetic fields, Phys. Rev. B 73, 235306 (2006).

